# A Mobile Phone App to Support Adherence to Daily HIV Pre-exposure Prophylaxis Engagement Among Young Men Who Have Sex With Men and Transgender Women Aged 15 to 19 Years in Thailand: Pilot Randomized Controlled Trial

**DOI:** 10.2196/25561

**Published:** 2022-04-21

**Authors:** Surinda Kawichai, Wipaporn Natalie Songtaweesin, Prissana Wongharn, Nittaya Phanuphak, Tim R Cressey, Juthamanee Moonwong, Anuchit Vasinonta, Chutima Saisaengjan, Tanat Chinbunchorn, Thanyawee Puthanakit

**Affiliations:** 1 Center of Excellence for Pediatric Infectious Diseases and Vaccines Chulalongkorn University Bangkok Thailand; 2 Center of Excellence in Transgender Health Chulalongkorn University Bangkok Thailand; 3 Institute of HIV Research and Innovation Bangkok Thailand; 4 The Program for HIV Prevention and Treatment/ Unité Mixte de recherches Internationale 174 Faculty of Associated Medical Sciences Chiang Mai University Chiang Mai Thailand; 5 Department of Molecular and Clinical Pharmacology University of Liverpool Liverpool United Kingdom; 6 Focal Intelligence Co Ltd Bangkok Thailand; 7 Department of Pediatrics, Faculty of Medicine, Chulalongkorn University Bangkok Thailand

**Keywords:** mHealth, PrEP adherence, adolescents, men who have sex with men, transgender women, mobile phone

## Abstract

**Background:**

Widespread smartphone use provides opportunities for mobile health HIV prevention strategies among at-risk populations.

**Objective:**

This study aims to investigate engagement in a theory-based (information–motivation–behavioral skills model) mobile phone app developed to support HIV pre-exposure prophylaxis (PrEP) adherence among Thai young men who have sex with men (YMSM) and young transgender women (YTGW) in Bangkok, Thailand.

**Methods:**

A randomized controlled trial was conducted among HIV-negative YMSM and YTGW aged 15-19 years initiating daily oral PrEP. Participants were randomized to receive either youth-friendly PrEP services (YFS) for 6 months, including monthly contact with site staff (clinic visits or telephone follow-up) and staff consultation access, or YFS plus use of a PrEP adherence support app (YFS+APP). The target population focus group discussion findings and the information–motivation–behavioral skills model informed app development. App features were based on the *3Rs*—risk assessment of self-HIV acquisition risk, reminders to take PrEP, and rewards as redeemable points. Dried blood spots quantifying of tenofovir diphosphate were collected at months 3 and 6 to assess PrEP adherence. Tenofovir diphosphate ≥350-699 fmol/punch was classified as fair adherence and ≥700 fmol/punch as good adherence. Data analysis on app use paradata and exit interviews were conducted on the YFS+APP arm after 6 months of follow-up.

**Results:**

Between March 2018 and June 2019, 200 participants with a median age of 18 (IQR 17-19) years were enrolled. Overall, 74% (148/200) were YMSM; 87% (87/100) of participants who received YFS+APP logged in to the app and performed weekly HIV acquisition risk assessments (log-in and risk assessment [LRA]). The median duration between the first and last log-in was 3.5 (IQR 1.6-5.6) months, with a median frequency of 6 LRAs (IQR 2-10). Moreover, 22% (22/100) of the participants in the YFS+APP arm were frequent users (LRA≥10) during the 6-month follow-up period. YMSM were 9.3 (95% CI 1.2-74.3) times more likely to be frequent app users than YTGW (*P*=.04). Frequent app users had higher proportions (12%-16%) of PrEP adherence at both months 3 and 6 compared with infrequent users (LRA<10) and the YFS arm, although this did not reach statistical significance. Of the 100 participants in the YFS+APP arm, 23 (23%) were interviewed. The risk assessment function is perceived as the most useful app feature. Further aesthetic adaptations and a more comprehensive rewards system were suggested by the interviewees.

**Conclusions:**

Higher rates of PrEP adherence among frequent app users were observed; however, this was not statistically significant. A short app use duration of 3 months suggests that they may be useful in establishing habits in taking daily PrEP, but not long-term adherence. Further studies on the specific mechanisms of mobile phone apps that influence health behaviors are needed.

**Trial Registration:**

ClinicalTrials.gov NCT03778892; https://clinicaltrials.gov/ct2/show/NCT03778892

## Introduction

### Background

The emergence of advanced technologies and widespread use of smartphones provides opportunities for new HIV research and prevention strategies using mobile health (mHealth) within high-risk populations [1,2]. mHealth is the practice of medical and public health supported by mobile devices, such as mobile phones, patient monitoring devices, PDAs, and other wireless devices [3]. Data on user interaction with web-based intervention tools are readily available as part of such technologies and form useful surrogate markers of user engagement characteristics [4]. Despite the high potential for mHealth use in service delivery, there is a lack of data on how this is best delivered in low- and middle-income countries and in youth [1,2].

Men who have sex with men (MSM) and transgender women (TGW) are at high risk for HIV infection globally [5]. In Thailand, over 80% of new infections occur in this population [6], with young MSM (YMSM) and young transgender women (YTGW) at even greater risk than their older counterparts [7,8], as HIV incidence among YMSM and YTGW is 4-12 per 100 person-years [9-13], well above the 3 per 100 person-years World Health Organization incidence recommendation priority for offering HIV pre-exposure prophylaxis (PrEP) [14].

With the safety and effectiveness of PrEP established [14-18], the current challenge with PrEP is how best it can be implemented in key populations. The use of PrEP to prevent HIV has been recommended in the Thai national guidelines since 2014 [19], initially available as a fee-based PrEP the same year and also the following year through PrEP demonstration projects in 2015. The Princess PrEP Project, a key population-led PrEP service delivered to MSM and TGW by lay providers, was launched in 2016, contributing to 60% of all national PrEP uptakes [20,21]. *PrEP2start* was then launched by the Thai Ministry of Public Health in 2017, a nationwide scale-up initiative to increase accessibility to PrEP for key populations, including TGW and MSM [22]. Most recently, in 2019, PrEP became available under Thailand’s Universal Health Coverage Scheme [21].

Given that PrEP efficacy is highly reliant on adherence [23], strategies to support adherence, encompassing those from biological, psychological, and social fronts, are key to its effectiveness at the public health level [24-27]. PrEP adherence in adolescents is known to be a challenge with adherence observed at 28% to 48% after 6 months of use, and it has been acknowledged that tailored approaches for this key population are needed to deliver effective prevention programs [25-28]. mHealth has been implemented in managing various health behaviors in adolescents, including sexual health promotion, disease prevention, antiretroviral adherence in HIV, and emotional health support, and outcomes have been promising [29,30]. A pilot mHealth study, iText, to support PrEP adherence motivation using weekly SMS text or email support messaging, found that it was acceptable, particularly among young participants, and demonstrated a 50% to 77% reduction in missed PrEP dosing with its use [31]. There is currently a lack of data evaluating eHealth service delivery strategies from low- to middle-income countries to support HIV prevention efforts [1,30,32]. Mobile phone health apps that use bidirectional interactions and are designed based on behavioral theories have been found to be more effective in influencing health behaviors than those that send unidirectional messages [2]. A frequently used theory in mobile app design is the information–motivation–behavioral (IMB) skills model that asserts that initiation and maintenance of health-promoting behaviors come about as a result of a combination of health-related IMB skills [2,33].

### Objectives

Our aim in this study is to investigate participant engagement and the impact on PrEP adherence of the *Project Raincoat* mobile phone app, which was based on the IMB skills model developed to support PrEP adherence among YMSM and YTGW at risk of HIV acquisition, in the context of an adolescent-friendly clinic providing bidirectional web-based communications in Thailand. The primary results of this trial showed no difference in measured PrEP adherence between arms that received the app and those that did not; approximately 50% of all PrEP users achieved protective drug levels and no seroconversions were observed [33].

## Methods

### Overview and Ethical Considerations

This was a prospective randomized controlled trial of oral daily PrEP in youth at risk of HIV acquisition in Bangkok, Thailand [34]. Adolescents aged 15-19 years, assigned male gender at birth, and self-defining as MSM or TGW with HIV risk acquisition behaviors, defined as having >1 sex partner and inconsistent or no condom use in the preceding 6 months were included in this study. Participants could be new, current, or former PrEP users. They were randomized (1:1) to receive oral daily tenofovir-disoproxil fumarate/emtricitabine provided by either youth-friendly services (YFS) only or YFS plus the use of the *Raincoat* app (YFS+APP). This study was conducted in two different settings: (1) medical center–based facilities at the Thai Red Cross AIDS Research Center (TRCARC), the largest voluntary HIV testing center of Thailand, and the King Chulalongkorn Memorial Hospital, a major teaching hospital in Bangkok and (2) key population-led community-based drop-in centers operating as satellite sites of TRCARC, namely, the Rainbow Sky Association of Thailand (RSAT) and Service Workers In Group (SWING), both located in Bangkok, Thailand. Care at the 2 different medical center–based facilities was performed by the same team that moved between the 2 locations and tended to receive clients slightly more medically informed than those in the community-based drop-in centers. RSAT, owing to its proximity to universities and nearby student accommodation, received proportionally more cases than SWING; however, staff at both RSAT and SWING delivered similar care, with both teams receiving training from TRCARC and our study team on standard operating procedures.

The institutional review board approval was granted by the Faculty of Medicine, Chulalongkorn University (number 1091/2017), with a waiver for parental consent. This study was registered with ClinicalTrials.gov (NCT03778892).

### Youth-Friendly Services

YFS included the following: (1) monthly engagement via either in-person clinic visits (months 1, 3, and 6) or telephone calls (months 2, 4, and 5) and (2) access to counselors and site staff outside scheduled visits through web-based messaging or telephone calls with responses provided within 24 hours. Clinic visits were available during weekdays and Saturday mornings to accommodate adolescent lifestyles. Motivational interviewing focused on HIV risk reduction and empowerment on using available HIV prevention methods was used by counselors during all interactions with clients [35,36].

### Mobile Phone App Development and Use

The *Raincoat* mobile app was developed in conjunction with Focal Intelligence Co. Ltd. and designed using the IMB skills model guided by input from two adolescent focus group discussions (FGDs), one with 3 MSM and one with 3 TGW, all aged between 15 and 19 years [37,38]. Discussions were conducted using a semistructured interview guide with topics on desirable app functions, aesthetic preferences, potential barriers, and motivators for use. Key themes and subthemes were identified from content analysis, which then informed app design. The app prototype was tested with staff providing HIV prevention care to clients at all study sites (2 from each site, totaling 8 usability testers) whose feedback was used to inform the final app design.

The final app designed had three main features, which we refer to as the *3Rs*: *risk assessment*, *reminders*, and *rewards*. The former addresses the *information* component of the IMB model, and the latter two address the *motivation* component, with further explanation as follows: (1) *risk:* self-assessment of HIV acquisition risk with a once-weekly data input portal on the number of sex acts, sex partners, PrEP pills taken, and condom use, which was then used to calculate a feedback HIV risk level of low, medium, high, and very high; (2) *reminders:* in-built alarms for taking PrEP and HIV risk self-assessment using default set messages that were customizable; and (3) *rewards:* points were rewarded in real time for data input (maximum reward of 21 points per week) as well as to responding to staff follow-up calls (5 points each), attendance of clinic visits (10 points each), and negative anti-HIV test results (50 points each). Points rewarded were part of the intervention and were available to the YFS+APP arm only. Points were exchangeable for cash, with redemption being available at every 100 points if redeemed before the end of the study. Moreover, 100 points were exchangeable for ฿100 (US $3) at month 3 or month 6 clinic visits. A maximum of 719 points could be accumulated when using the app for 6 months.

The app was available only on the Android operating system. Android mobile phones were available for loans to those using other operating systems. Participants were free to log in to the app at any time. Staff provided guidance at their enrollment visit on how to load the app and introduced its functions. Participants required an email for initial registration and were asked to create their own usernames and passwords. Usernames and passwords were required for each log-in to ensure confidentiality. If participants had any issues using the app, they were free to contact the staff to resolve them. Customizable medication reminder messages could be created and changed at any time. The app was accessible offline for all features apart from the self-risk assessment feature, which could only be used with internet connectivity.

### Mobile App Users—Exit Interviews

To solicit the attitudes of users toward the app, exit interviews were conducted using open-ended questions. Using convenience sampling, we interviewed a selection of participants who had (1) never logged in to the app, (2) used the app infrequently (defined as performance of log-in and risk assessment [LRA] of 1-9 times throughout the trial), and (3) been frequent users (defined as LRA≥10 times throughout the trial), with at least 10% of the total interviewees sampled from each group. Interview topics included facilitators and barriers to app use, valuable features of the app, reasons for nonuse, and general suggestions for improvement.

### Study Procedures and Follow-up of PrEP Services

Follow-up clinic visits occurred at months 1, 3, and 6, and telephone contact was made at months 2, 4, and 5. Risk behaviors and risk perception surveys were conducted for each monthly contact.

Substance use information was collected from self-reported surveys completed by participants privately via an electronic form consisting of alcohol, sildenafil citrate, and other recreational drugs (eg, amphetamines, methamphetamines, ketamine, poppers [volatile alkyl nitrates], and marijuana). HIV blood testing was performed at baseline and at months 1, 3, and 6.

### Biological Measurement of PrEP Adherence

Dried blood spots (DBSs) were collected for quantification of tenofovir diphosphate (TFV-DP) concentrations at months 3 and 6 of follow-up. Whole blood samples were collected on Whatman Protein Saver 903 cards. DBSs were stored at −70 °C until analysis. TFV-DP was analyzed by liquid chromatography mass spectrometry. The TFV-DP calibration curve range was 200-10,000 fmol/3 mm punch [23,39] performed at the Program for HIV Prevention and Treatment Research Institute for Sustainable Development Pharmacology Laboratory at the Faculty of Associated Medical Sciences, Chiang Mai University. Details of the study methods have been previously published [34].

### Statistical Analysis

Data on app use were downloaded for analysis after 6 months of follow-up. App paradata collected included first and last log-in dates, number of times logged in, number of LRAs, use of reminder messages, use of the medication alarm function, and total points accumulated.

The number of LRAs was used as an independent variable to assess user engagement with the app. TFV-DP DBS concentrations were used to evaluate PrEP adherence, with TFV-DP levels ≥700 fmol/punch considered good adherence (equivalent to ≥4 tablets/week) [23].

Continuous variables were presented as means with SDs or medians with IQRs, and categorical variables with absolute numbers and percentages. The chi-square test, Z test, Fisher exact test, odds ratios, and 95% CIs were used for group comparisons and associations as appropriate. Stata/SE (version 13.0; StataCorp) was used for quantitative data analyses.

## Results

### Overview

Details of baseline characteristics and comparisons between the control arm (YFS) and the intervention arm (YFS+APP) have previously been published [34]. In brief, between March 2018 and June 2019, 200 HIV-negative participants were enrolled, 100 received YFS and 100 YFS+APP. The median age at enrollment was 18 (IQR 17-19) years, with 73.5% (147/200) self-defining as YMSM and 26.5% (53/200) as YTGW. Participants were enrolled and followed-up at medical center–based facilities (135/200, 67.5%) or community-based drop-in centers (65/200, 32.5%). Baseline characteristics between the YFS and YFS+APP arms were similar except for self-reported substance use in the preceding 3 months, which was higher in the YFS+APP arm, 18% (18/100), than in the YFS arm, 8% (8/100; *P*=.04) [34]. Of the 143 adolescents reporting sexual activity in the past month, 94 (65.7%) reported inconsistent condom use. Of the 200 participants, 187 (93.5%) rated themselves as having a low risk of HIV acquisition. There were no significant differences in PrEP adherence between the YFS and YFS+APP arms. PrEP adherence was 51% (40/79) in the YFS arm and 54% (44/81) in the YFS+APP arm (*P*=.64) at month 3 and was 44% (30/68) in the YFS arm and 49% (36/73) in the YFS+APP arm (*P*=.54) at month 6, further details of which have previously been published [34].

### Risk Assessments

**Figure 1:**
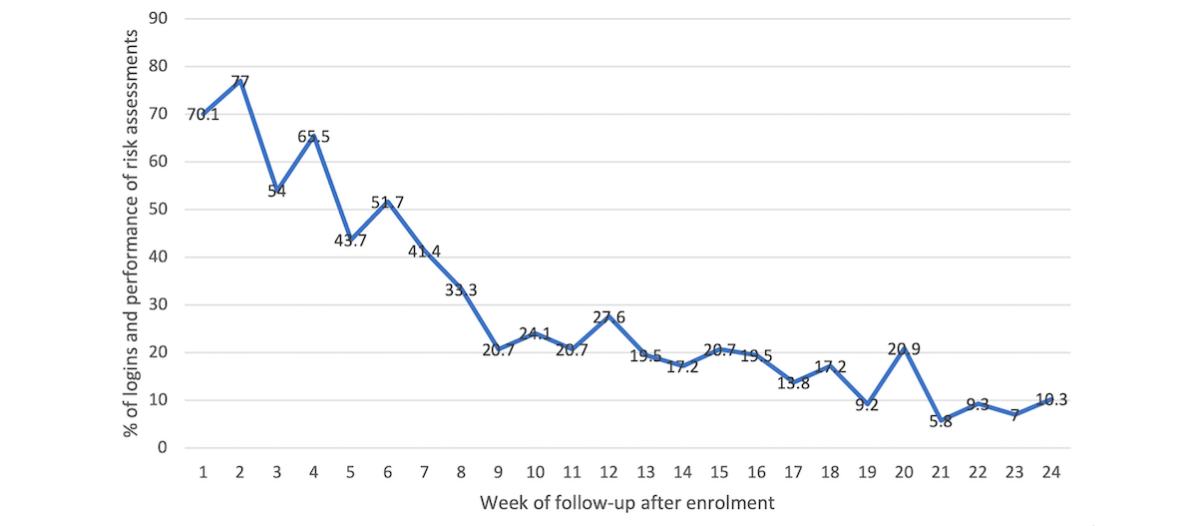
Percentage of participants who logged in and performed risk assessments by week after enrollment (N=87).

Of the 100 participants randomized to receive YFS+APP, 87 (87%) used LRA at least once during the follow-up period. Of these 87 app users, 55 (63%) used their own phones, and the remaining used loaned Android operating system phones. Of the 13 participants who never logged into the app, 10 (77%) used their own phones and 3 (23%) used loaned phones. Among the app users, the median (IQR) duration between the first and last LRA was 3.5 (1.6-5.6) months, with a median LRA frequency of 6 (IQR 2-10). There was no difference in median LRA frequency between participants who used their phones and those who used loaned phones (*P*=.21). The percentage of participants who used LRA declined over time, that is, 77%, 52%, and 28% at 2, 6, and 12 weeks after enrollment, respectively (Figure 1)**.**

Of the 87 app users, 65 (76%) were infrequent users (LRA<10). Associations of LRA frequency and baseline characteristics among app users were assessed, and only gender identity was significantly associated with LRA, with YMSM 9.3 times (95% CI 1.2-74.3; *P*=.04) more likely to be frequent users than YTGW, but with a very wide CI (Table 1).

Associations between LRA frequency and PrEP adherence determined by TFV-DP DBS levels at months 3 and 6 are shown in Table 2.

**Table 1:** Baseline characteristics of participants in the YFS+APP^a^ arm who logged in to the mobile phone app and performed risk assessment (LRA^b^) at least once during the trial follow-up period and associations with LRA.

Demographic characteristics		Total (n=87), n (%)	LRA≥10 times, n (% by characteristics)	OR^c^ (95% CI)	*P* value
**Gender identity**					
	MSM^d^	66 (75)	21 (31.8)	9.3 (1.2-74.3)	.04
	TGW^e^	21 (24)	1 (4.8)	1	N/A^f^
**Age at enrollment (years)**					
	15-17	29 (33)	6 (20.7)	1	N/A
	18-19	58 (66)	16 (27.6)	1.5 (0.5-4.2)	.49
**Number of sex partner or partners in the last month**					
	0	25 (28)	8 (32)	1	N/A
	1	38 (43)	12 (31.5)	1.0 (0.3-2.9)	.97
	≥2	24 (27)	2 (8.3)	0.2 (0.0-1.0)	.05
**Condom use in the past month among participants reporting sexual activity (n=62)**					
	100	18 (29)	4 (22.2)	1	N/A
	<100	44 (71)	10 (22.7)	1.0 (0.3-3.8)	.97
**Recreational substance use in the past 3 months (n=62)**					
	No	70 (80)	19 (27.1)	1	N/A
	Yes	17 (19)	3 (17.6)	0.6 (0.2-2.2)	.42
**Self-perceived risk (n=62)**					
	Low or moderate risk	52 (59)	16 (30.8)	1	N/A
	High risk	35 (40)	6 (17.1)	0.5 (0.2-1.34)	.16

^a^YFS+APP: youth-focused strategies plus mobile phone app.

^b^LRA: log-in and risk assessment.

^c^OR: odds ratio.

^d^MSM: men who have sex with men.

^e^TGW: transgender women.

^f^N/A: not applicable (reference).

**Table 2:** Associations of Raincoat mobile phone app use and HIV PrEP^a^ adherence (tenofovir diphosphate level ≥700 fmol/punch) at months 3 and 6.

App users			PrEP adherent, n (%; 95% CI)		Unadjusted				Adjusted^b^		
					Odds ratio (95% CI)		*P* value		Odds ratio^c^ (95% CI)		*P* value
**At 3 months**											
	YFS^d^ only (n=79)	40 (50; 39.6-61.6)		1.0		N/A^e^		N/A		N/A	
	YFS and infrequent app use (n=53)	29 (55; 41.3-68.1)		1.8 (0.6-2.4)		.64		1.1 (0.5-2.4)		.84	
	YFS and frequent app use (n=21)	14 (67; 46.5-86.8)		2.0 (0.7-5.4)		.19		1.8 (0.6-5.4)		.59	
**At 6 months**											
	YFS only (n=68)	30 (44; 32.3-55.9)		1.0		N/A		N/A		N/A	
	YFS and infrequent app use (n=47)	21 (45; 30.5-58.9)		1.0 (0.5-2.2)		.95		0.8 (0.4-1.9)		.64	
	YFS and frequent app use (n=22)	13 (59; 38.5-79.6)		1.8 (0.7-4.8)		.22		1.8 (0.6-5.3)		.27	

^a^PrEP: pre-exposure prophylaxis.

^b^Adjusted by gender identity (men who have sex with men vs transgender women), age at enrollment (15-17 vs 18-19) (years), number of sex in the past month at enrollment interviewed (0 vs 1 and ≥2), and self-perceived HIV acquisition risk at enrollment.

^c^Unadjusted odds ratio (95% CI) of adjusted variables in footnote^a^, as previously published [34].

^d^YFS: youth-friendly services.

^e^N/A: not applicable (reference).

The overall percentage of PrEP adherence was higher in frequent app users than in infrequent app users and the YFS arm, but this did not reach statistical significance. At month 6, the proportion of participants who achieved TFV-DP≥700 fmol/punch was 59% (13/22), 44% (30/68) among frequent app users, 45% (21/47) for infrequent users, and 44% (30/68) in the YFS arm (*P*=.47).

### Exit Interview Findings

A total of 23 adolescents in the YFS+APP arm participated in exit interviews, of which 18 (78%) were YMSM and 20 (87%) were active PrEP users at the time of interview. Of all 23 participants interviewed, 3 (13%) had never logged in to the app, 17 (74%) were infrequent users, and 3 (13%) were frequent users. Interviews were conducted at a median (IQR) of 8.5 (5.0-12.4) months after completion of the study.

Reasons for app nonuse were categorized into those that were app-specific and those that were lifestyle-related. App-specific factors included the inconvenience of using a borrowed phone, requiring email verification during the registration process, finding app aesthetics and functions unengaging, and its lack of complete offline functionality. Lifestyle factors included changing phones, not loading the app onto their new phone, and being too busy with work and studies to use the app.

Regarding the risk assessment app feature, participants liked this feature the most because of the colorful graphics used in feeding back risk levels, because of not requiring the answering of too many questions, and also because it helped them to be more aware of their HIV risk. All 20 app user interviewees had used risk assessment features and reported that they performed risk assessments in the beginning and less later on for multiple reasons, including not being sexually active during the time, shift work, being busy with studies, forgetting to take their phone with them, or just forgetting to do the assessment.

Interviewees emphasized that for an app to be desirable for adolescents, it would need to be easy to use, feature attractive aesthetic features such as customizable color schemes and customizable avatars, and also provide the opportunity to interact with others. Privacy was also another major concern, and passcode protection and discreet branding of the app were also important. Although many participants expressed a desire for the app to have more information owing to its convenience and credibility, some felt that searching for information themselves on the web was easier and also provided the most up-to-date information. The app also needed to be competitive with other available apps, both health-related and nonhealth-related, in the market.

### Reminders for Taking PrEP and Visit Reminders

Of the 87 app users, 45 (52%) used PrEP reminders and 34 (39%) clinic appointment reminders. Of the 45 participants who used PrEP reminders, 8 (18%) customized their reminder messages. Proportions of PrEP adherence observed between those who used and did not use the PrEP reminder function did not differ at month 3 (50% vs 68%; *P*=.13) or month 6 (50% vs 53%; *P*=.82).

Of the 20 app user exit interviewees, 15 (75%) reported using the PrEP reminder feature, and of the 15 reminder users, 8 (53%) liked the function because it helped them take PrEP on time. Moreover, 1 participant viewed this function as the most important feature of the app. Some interviewees who stopped using this function said they switched to using their phone alarm as it was more convenient, particularly in those using a borrowed study phone, as sometimes the app alarm did not go off as set.

### Rewards and Redemption

Of the 87 app users, 51 (59%) accumulated sufficient points for cash redemption (range 100-504 points). Among the users, 49% (43/87) earned 100 to 199 points, 33% (29/87) earned 200 to 299 points, 16% (14/87) earned 300 to 399 points, and 2% (2/87) earned ≥400 points.

For redeeming prizes, exit interviewees, with 65% (13/20) of them having had participated in cash redemption, wanted the entire process to be automated (although accumulated points were visible in the app, redemption had to be done manually at clinic visits by study staff). The cash rewards used in this app were liked by 25% (5/20) of the app users, but many felt there was a need to increase variation in rewards with additional gimmicks, such as exchange of points for other prizes such as movie tickets and food vouchers. More importantly, to motivate the use of the app, the timing of reward redemption they felt should be earlier than at the 3- and 6-month clinic visits in this study.

### Engagement With Site Staff Outside Monthly Clinic or Phone Call Visits

There were 578 interactions between study staff and 140 participants, with a median (IQR) of 4 (3-5) times per person. Engagement with staff was similar between the YFS (67/100, 67%) and YFS+APP (73/100, 73%) arms. Of the 578 interactions, 299 (51.7%) were initiated by clients. The median (IQR) time to first contact after enrollment was 6 (3-13) weeks. The topics of interactions included making appointments (128/299, 42.8%), relationships and personal issues (70/299, 23.4%), PrEP inquiries (54/299, 18.1%), HIV risk (29/299, 9.7%), and sexually transmitted disease diagnosis and management (18/299, 6%; Table 3).

Regarding what service aspects would keep them coming back, many felt this ultimately came down to staff being friendly, open, approachable, reliable, understanding, and holistic in their care.

**Table 3:** Topics of inquiry initiated by participants to site staff during 6 months of follow-up.

Contact topics	Participants initiating contact (n=140^a^), n (%)^b^	Contacts (n=299), n (%)	YFS^c^ (n=149), n (%)	YFS+APP^d^ (n=150), n (%)	*P* value
Clinic appointments	90 (64.3)	128 (42.8)	64 (43)	64 (42.7)	.96
Relationships and personal issues	62 (37.1)	70 (23.4)	28 (18.8)	42 (28)	.06
HIV PrEP^e^	44 (31.4)	54 (18.1)	34 (22.8)	20 (13.3)	.03
HIV risk acquisition	26 (18.6)	29 (9.7)	14 (9.4)	15 (10)	.86
Sexually transmitted diseases	11 (7.8)	18 (6)	9 (6)	9 (6)	.99

^a^Total number of participants initiating contact.

^b^Some participants inquired more than once on >1 topic.

^c^YFS: youth-friendly services.

^d^YFS+APP: youth-friendly services plus mobile phone app.

^e^PrEP: pre-exposure prophylaxis.

## Discussion

### Principal Findings

This randomized controlled study examined how a smartphone PrEP adherence support app used among adolescents in Thailand affected PrEP adherence and observed higher proportions of PrEP adherence among frequent app users than among infrequent users and the YFS arm at both months 3 and 6, although this did not reach statistical significance. To our knowledge, this study is one of the few studies that address key populations where a dearth of information exists in both young adolescents and low- and middle-income settings to inform future policy and practices in such settings [2,30,40]. Although some degree of success in using telehealth at public health levels in Thailand has been seen in some studies in Thailand, including the use of mobile phone SMS text messaging to support PrEP adherence [41], telephone-based drug adherence support for tuberculosis [42], web-based recruitment and linkage to home-based HIV testing [43], the use of mHealth technologies in HIV prevention efforts in Thailand and the Asia Pacific region is in its early stages, and few publications beyond preimplementation studies exist regarding mobile phone app implementation and effectiveness in risk behavior modification. The app produced and used in this trial was based on a behavioral change theory, IMB, where *information* provided on risk with self-assessments and *motivation* with reminders with medication alarms and point rewards.

### Risk Assessment

Of the 87 participants, 22 (25%) were classified as frequent users, reflecting that app use was short-term in most users, which is consistent with a previous observation that 33% of users stop using their device in <6 months and 39% of commercial health apps were used ≤10 times before use was discontinued [44]. The lack of additional PrEP adherence benefit seen with mobile phone app use in this trial may have been due to *dilution* by the large proportion of infrequent app users and nonapp users in those randomized to use it. Given that it is known that, globally, mobile app retention, defined as the use of an app in the preceding 7 days, is only approximately 15%, the challenge of measuring real-world mobile phone app effectiveness could be addressed with larger trials to establish whether this short duration of use is sufficient to elicit behavioral change or whether strategies are needed to encourage longer use to enable behavioral change [45]. It is also important to consider whether app use is able to elicit behavioral change, specifically by what mechanism, which could be done by isolation and testing of individual components to allow future innovations to build upon in developing effective strategies, currently a research gap in mHealth technologies [32,44,46]. Improvement of aesthetics and ensuring features were customizable, having more options for cash reward exchanges, having more social interaction, and more health information were also major themes that arose in the exit interviews as areas in which users felt the app could be improved to increase user engagement. This is consistent with a previous review of mobile phone app use in HIV prevention that app inclusiveness and interactivity are more likely to attract and retain users as well as elicit behavioral change [45].

Individuals may have felt no need to continue performing self-risk assessments if they intended to continue the same preventive measures. Those who continued with LRA may have been those who had fluctuations in sexual HIV risk behaviors, the *worried well*, or point reward collectors. Many exit interviewees said that the action of assessment was a reminder to consider self-risk, which in turn influenced planned health behaviors. We feel this self-acknowledgment of one’s true risk level was supported by the app, which enabled privacy and discrete assessment and bypassed the need for human interaction and potential judgmental attitudes faced in making this assessment. In addition, users were able to try inputting data relevant to different scenarios, as it was possible to repeat assessments in planning upcoming sexual encounters. We think that the large drop-off of users toward the end of the trial is reflective of findings from previous studies that apps are mainly effective in those who are already motivated but are less effective at supporting user capabilities and maintaining behavioral changes [46].

On the basis of the exit interview findings, the *Raincoat* app was found to be easy to use. App design allowing ease of utility clearly influenced how likely participants would use the app. This observation is in keeping with previous studies that found simple interfaces may reduce the time participants need to spend on the app and therefore improve retention [30].

### Reminders

Over half of the app users used their PrEP reminder function. Our exit interviews found that many users felt that the function was very useful for supporting PrEP adherence. Tailored, short pieces of information on self-risk fed back at weekly intervals used in this trial has been reported to be the preferred mode of messaging in other studies compared with general health information [46]. However, in this study, no association was observed between medication alarm use and PrEP adherence, suggesting that this feature alone may not support PrEP adherence for all users. Furthermore, given that only 18% (8/45) of participants customized their reminder message function, it could be argued that this is a nonessential feature for future versions. All those interviewed said that the alarm function did not always work in this trial was a barrier to their engagement with it. This study highlighted the implementation of mHealth technologies, particularly in low- and middle-income settings. Plans and budget allocations must be made for the technical maintenance of their functionality to ensure engagement and maximum benefit of use over time. It should also be noted that some participants switched to using their own phone alarm for medication reminders, suggesting that the alarm function may not be an essential in-app feature for adolescents, particularly in resource-limited settings.

### Rewards and Redemption

In this study, we observed that cash rewards may not be sufficient on their own to influence health behaviors, which is consistent with a previous review that suggested, at best, conditional economic incentives can be a valuable part of but cannot replace other HIV prevention approaches and, importantly, need to be adapted to specific contexts and populations for maximal success [47]. This would need to be carefully experimentally isolated to explore this possibility further [48].

### Engagement With Site Staff

Engagement in this study at monthly scheduled visits or phone calls plus optional additional staff consultation producing a 73% retention rate may have reduced the impact of receiving the app intervention seen, given that it is known that 2-way interaction between providers and clients is known to improve medication adherence [2]. Appointment-related queries made up nearly half of all contact topics in both study arms and accounted for 64.3% (90/140) of all participants who initiated contact. This strongly suggests that an automated system in the app to book and confirm appointments would improve convenience for both service users and providers. Relationships and personal issues and PrEP-related queries also made up approximately one-fifth each of queries to staff, suggesting that specific in-app information provision and staff training on these issues may benefit service users. These data highlight the unique considerations to be made in adolescent and young adult HIV prevention service provision to address developmental needs to support their decision-making, establishment of identity, interpersonal challenges, and mental health issues commonly seen at this stage of life [27].

### Types of Clinic Settings or Services

Although the intervention of interest was a mobile phone app, it must be acknowledged that the contexts in which this study occurred were medical center–based facilities with HIV expertise or community-based organizations (CBOs), both of which are unique to general PrEP provision services available in Thailand. All the locations used in this study have study staff with training on the same basis to deliver the intervention and a focus of work on HIV prevention and client-centered care, with medical center–based facilities having the strength of delivering the intervention directly and CBOs having the advantage of key population-led care, which, as has been seen in other studies, supported PrEP adherence and retention in services well [20,49]. It is possible that such differences could have acted as effect modifiers in the interventions studied. General PrEP services in Thailand are delivered at medical center–based facilities with a larger variety of health issues and have a larger volume of service users, making holistic health care much more challenging to deliver. Medical centers and CBOs may draw different types of populations, and with randomization, the characteristics are balanced between the 2 arms. The heterogeneity of the sample makes the generalizability of the study results to broader populations.

It is also recognized that mHealth has greatly facilitated the maintenance of some health care delivery services in the last year during the COVID-19 pandemic [50-53]. Evidence suggests that HIV at-risk populations globally, including YMSM and YTGW, have faced considerable health care access disruptions and urgent development and implementation of telehealth as part of efforts to minimize such disruptions, in addition to other policy level initiatives, will minimize associated care disruption-related HIV morbidity and mortality [54,55]. The largest PrEP provider of Thailand, the *Princess PrEP Program*, has recognized since its initiation in 2016 that service users require adherence and retention support, particularly in TGW, which could be supported using mHealth technologies [21].

### Limitations

Although the original design of this study was fully powered to compare those who received YFS versus YFS+APP, as a significant proportion of study participants infrequently used the app, it became necessary for the final analysis to stratify outcome groups into frequent users (LRA10) and infrequent users (LRA<10). The actual analysis was therefore not fully powered; thus, results from this study should be viewed as preliminary as part of a pilot randomized control trial. Another limitation of this study was that the app used for the intervention in this study was based on just 2 FGDs, which may have provided an inadequate range of views in the design of the app. In addition, the protocol of this study was designed to describe the *natural history* of app use without any formal encouragement from the study team. App paradata were therefore collated at the end of study for this analysis. However, another possible policy that could have been taken was to continually look at app use and discuss app use obstacles at each contact to rapidly assess and address functionality problems and also to collect design issues *real time* when these issues are *fresh* in the minds of the users and more likely to be recalled more clearly. In addition, given that the efficacy of mobile technologies is also linked to literacy and the performance of this study in a large city, findings from it may not apply to a more rural area where access to mobile technologies and literacy may be more challenging and less benefit gained from such a form of service provision enhancement, a limitation of mHealth seen previously [44]. Only a total of 6 interviewees participated in FGDs that informed the design of this app, which may have affected the generalizability of the preference representation drawn from this sample of adolescents. The sampling frame used for exit interviews in this study was also biased toward those who were classified as frequent users of the app, which may have led to findings being more biased toward favoring app functions. Given no differences were seen between arms, interviews with control arm participants could have been done to provide further information on possible reasons for this. Interviews in this study were conducted for quite a long time after study completion in most cases, which may have led to limited data quality owing to implications on participant recall. The small sample size of this study also limits the generalizability of the conclusions drawn from this study, and it is possible that the intervention was not efficacious or the sample size was too small to see any effect present.

### Conclusions

Using a target population-informed design for a mobile phone app supporting PrEP adherence in an adolescent PrEP implementation study, we observed higher rates of PrEP adherence among those who frequently used the app, although the difference was not statistically significant. App use dropped by 50% by week 6, suggesting its possible usefulness in establishing habits but not for long-term adherence maintenance. Further studies on specific mechanisms in which mobile apps are effective in influencing health behaviors are needed to inform future mHealth scale-up operations, particularly in low- and middle-income settings with limited staff service capacity. mHealth development and implementation will ultimately benefit health access and delivery for HIV prevention services beyond its current apps in the current COVID-19 pandemic.

## Appendix

Multimedia Appendix 1 CONSORT eHEALTH Checklist (V 1.6.1).

## Abbreviations

CBO: community-based organization
CIPHER: Collaborative Initiative for Pediatric HIV Education and Research
DBS: dried blood spot
FGD: focus group discussion
IMB: information–motivation–behavioral
LRA: log-in and risk assessment
MSM: men who have sex with men
PrEP: pre-exposure prophylaxis
RSAT: Rainbow Sky Association of Thailand
SWING: Service Workers in Group
TFV-DP: tenofovir diphosphate
TGW: transgender women
TRCARC: Thai Red Cross AIDS Research Center
YFS: youth-friendly services
YFS+APP: youth-friendly services plus mobile phone app
YMSM: young men who have sex with men
YTGW: young transgender women
