# Supplementary material for: A Mobile Phone App to Support Adherence to Daily HIV Pre-exposure Prophylaxis Engagement Among Young Men Who Have Sex With Men and Transgender Women Aged 15 to 19 Years in Thailand: Pilot Randomized Controlled Trial
Source: JMIR Mhealth Uhealth. 2022 Apr 21;10(4):e25561. doi: 10.2196/25561 (PMC9073624; doi:10.2196/25561)
Supplement: Multimedia Appendix 1 [file mhealth_v10i4e25561_app1.pdf]

## CONSORT-EHEALTH Checklist V1.6.2 Report	Manuscript Number	25561
**(based on CONSORT-EHEALTH V1.6), available at [http://tinyurl.com/consort-ehealth-v1-6].**		
**Date completed**		
4/17/2022 2:52:15		
**by**		
Wipaporn Natalie Songtaweesin		
A Mobile Phone App to Support Adherence to Daily HIV Pre-Exposure Prophylaxis Engagement Among Young Men Who Have Sex With Men and Transgender Women Aged 15 to 19 Years in Thailand: Pilot Randomized Controlled Trial		
TITLE		
**1a-i) Identify the mode of delivery in the title**		
"A Mobile Phone App to Support Adherence to Daily HIV Pre-Exposure Prophylaxis Engagement Among Young Men Who Have Sex With Men and Transgender Women Aged 15 to 19 Years in Thailand: Pilot Randomized Controlled Trial"		
**1a-ii) Non-web-based components or important co-interventions in title**		
**1a-iii) Primary condition or target group in the title**		
"Young Men Who Have Sex With Men and Transgender Women Aged 15 to 19 Years in Thailand"		
ABSTRACT		
**1b-i) Key features/functionalities/components of the intervention and comparator in the METHODS section of the ABSTRACT**		
"Participants were randomized to receive either youth-friendly PrEP services (YFS) for 6 months, which included monthly contact with site staff (clinic visits or telephone follow-up) and staff consultation access, or YFS plus use of a PrEP adherence support app (YFS+APP). The target population focus group discussion findings and the information–motivation–behavioral skills model informed app development. App features were based on the 3Rs, namely, risk assessment of self-HIV acquisition risk, reminders to take PrEP, and rewards as redeemable points."		
**1b-ii) Level of human involvement in the METHODS section of the ABSTRACT**		
The app was fully automated but care was supported by site staff in addition. Details of the app itself have previously been detailed in a previous publication so not mentioned in-depth for this paper.<br><br>"Participants were randomized to receive either youth-friendly PrEP services (YFS) for 6 months, which included monthly contact with site staff (clinic visits or telephone follow-up) and staff consultation access, or YFS plus use of a PrEP adherence support app (YFS+APP). The target population focus group discussion findings and the information–motivation–behavioral skills model informed app development. App features were based on the 3Rs, namely, risk assessment of self-HIV acquisition risk, reminders to take PrEP, and rewards as redeemable points."		
**1b-iii) Open vs. closed, web-based (self-assessment) vs. face-to-face assessments in the METHODS section of the ABSTRACT**		
**1b-iv) RESULTS section in abstract must contain use data**		
**1b-v) CONCLUSIONS/DISCUSSION in abstract for negative trials**		
INTRODUCTION		
**2a-i) Problem and the type of system/solution**		

This intervention was intended to be used as part of a broader health care program in young men who have sex with men and transgender women at risk of HIV<br><br>"Our aim in this study is to investigate participant engagement and the impact on PrEP adherence of the Project Raincoat mobile phone app, which was based on the IMB skills model developed to support PrEP adherence among YMSM and YTGW at risk of HIV acquisition, in the context of an adolescent-friendly clinic providing bidirectional web-based communications in Thailand."		
**2a-ii) Scientific background, rationale: What is known about the (type of) system**		
"PrEP adherence in adolescents is known to be a challenge with adherence observed at 28% to 48% after 6 months of use, and it has been acknowledged that tailored approaches for this key population are needed to deliver effective prevention programs [25-28]. mHealth has been implemented in managing various health behaviors in adolescents, including sexual health promotion, disease prevention, antiretroviral adherence in HIV, and emotional health support, and outcomes have been promising [29,30]. A pilot mHealth study, iText, to support PrEP adherence motivation using weekly SMS text or email support messaging, found that it was acceptable, particularly among young participants, and demonstrated a 50% to 77% reduction in missed PrEP dosing with its use [31]. There is currently a lack of data evaluating eHealth service delivery strategies from low- to middle-income countries to support HIV prevention efforts [1,30,32]. Mobile phone health apps that use bidirectional interactions and are designed based on behavioral theories have been found to be more effective in influencing health behaviors than those that send unidirectional messages [2]. A frequently used theory in mobile app design is the information–motivation–behavioral (IMB) skills model that asserts that initiation and maintenance of health-promoting behaviors come about as a result of a combination of health-related IMB skills [2,33]."		
**Does your paper address CONSORT subitem 2b?**		
"Our aim in this study is to investigate participant engagement and the impact on PrEP adherence of the Project Raincoat mobile phone app, which was based on the IMB skills model developed to support PrEP adherence among YMSM and YTGW at risk of HIV acquisition, in the context of an adolescent-friendly clinic providing bidirectional web-based communications in Thailand. The primary results of this trial showed no difference in measured PrEP adherence between arms that received the app and those that did not; approximately 50% of all PrEP users achieved protective drug levels and no seroconversions were observed [33]."		
METHODS		
**3a) CONSORT: Description of trial design (such as parallel, factorial) including allocation ratio**		
"This was a prospective randomized controlled trial of oral daily PrEP in youth at risk of HIV acquisition in Bangkok, Thailand [34]. "		
**3b) CONSORT: Important changes to methods after trial commencement (such as eligibility criteria), with reasons**		
Not applicable, no eligibility criteria were changed following commencement of the trial		
**3b-i) Bug fixes, Downtimes, Content Changes**		
**4a) CONSORT: Eligibility criteria for participants**		
"Adolescents aged 15-19 years, assigned male gender at birth, and self-defining as MSM or TGW with HIV risk acquisition behaviors, defined as having >1 sex partner and inconsistent or no condom use in the preceding 6 months were included in this study. Participants could be new, current, or former PrEP users. "		
**4a-i) Computer / Internet literacy**		
**4a-ii) Open vs. closed, web-based vs. face-to-face assessments:**		
Participants were recruited in-clinic (face-to-face), however information about the trial was available online.<br><br>" This study was conducted in two different settings: (1) medical center–based facilities at the Thai Red Cross AIDS Research Center (TRCARC), the largest voluntary HIV testing center of Thailand, and the King Chulalongkorn Memorial Hospital, a major teaching hospital in Bangkok and (2) key population-led community-based drop-in centers operating as satellite sites of TRCARC, namely, the Rainbow Sky Association of Thailand (RSAT) and Service Workers In Group (SWING), both located in Bangkok, Thailand."		
**4a-iii) Information giving during recruitment**		

**4b) CONSORT: Settings and locations where the data were collected**		
"This study was conducted in two different settings: (1) medical center–based facilities at the Thai Red Cross AIDS Research Center (TRCARC), the largest voluntary HIV testing center of Thailand, and the King Chulalongkorn Memorial Hospital, a major teaching hospital in Bangkok and (2) key population-led community-based drop-in centers operating as satellite sites of TRCARC, namely, the Rainbow Sky Association of Thailand (RSAT) and Service Workers In Group (SWING), both located in Bangkok, Thailand."		
**4b-i) Report if outcomes were (self-)assessed through online questionnaires**		
Some outcomes were measured through online questionnaires.<br><br>"The final app designed had three main features, which we refer to as the 3Rs: risk assessment, reminders, and rewards. The former addresses the information component of the IMB model, and the latter two address the motivation component, with further explanation as follows: (1) risk: self-assessment of HIV acquisition risk with a once-weekly data input portal on the number of sex acts, sex partners, PrEP pills taken, and condom use, which was then used to calculate a feedback HIV risk level of low, medium, high, and very high; (2) reminders: in-built alarms for taking PrEP and HIV risk self-assessment using default set messages that were customizable; and (3) rewards: points were rewarded in real time for data input (maximum reward of 21 points per week) as well as to responding to staff follow-up calls (5 points each), attendance of clinic visits (10 points each), and negative anti-HIV test results (50 points each). Points rewarded were part of the intervention and were available to the YFS+APP arm only. Points were exchangeable for cash, with redemption being available at every 100 points if redeemed before the end of the study. Moreover, 100 points were exchangeable for ฿100 (US $3) at month 3 or month 6 clinic visits. A maximum of 719 points could be accumulated when using the app for 6 months."<br><br>Other outcomes were assessed with exit interviews, clinic attendance, and biological measurements of drug levels in blood.		
**4b-ii) Report how institutional affiliations are displayed**		
**5) CONSORT: Describe the interventions for each group with sufficient details to allow replication, including how and when they were actually administered**		
**5-i) Mention names, credential, affiliations of the developers, sponsors, and owners**		
**5-ii) Describe the history/development process**		
**5-iii) Revisions and updating**		
**5-iv) Quality assurance methods**		
**5-v) Ensure replicability by publishing the source code, and/or providing screenshots/screen-capture video, and/or providing flowcharts of the algorithms used**		
**5-vi) Digital preservation**		
**5-vii) Access**		
Participants were provided the app in-clinic if they were randomised to receive the app, they did not have to pay to use the app.<br><br>"Participants could be new, current, or former PrEP users. They were randomized (1:1) to receive oral daily tenofovir-disoproxil fumarate/emtricitabine provided by either youth-friendly services (YFS) only or YFS plus the use of the Raincoat app (YFS+APP). "		
**5-viii) Mode of delivery, features/functionalities/components of the intervention and comparator, and the theoretical framework**		

"The Raincoat mobile app was developed in conjunction with Focal Intelligence Co. Ltd. and designed using the IMB skills model guided by input from two adolescent focus group discussions (FGDs), one with 3 MSM and one with 3 TGW, all aged between 15 and 19 years [37,38]. Discussions were conducted using a semistructured interview guide with topics on desirable app functions, aesthetic preferences, potential barriers, and motivators for use. Key themes and subthemes were identified from content analysis, which then informed app design. The app prototype was tested with staff providing HIV prevention care to clients at all study sites (2 from each site, totaling 8 usability testers) whose feedback was used to inform the final app design.<br><br>The final app designed had three main features, which we refer to as the 3Rs: risk assessment, reminders, and rewards. The former addresses the information component of the IMB model, and the latter two address the motivation component, with further explanation as follows: (1) risk: self-assessment of HIV acquisition risk with a once-weekly data input portal on the number of sex acts, sex partners, PrEP pills taken, and condom use, which was then used to calculate a feedback HIV risk level of low, medium, high, and very high; (2) reminders: in-built alarms for taking PrEP and HIV risk self-assessment using default set messages that were customizable; and (3) rewards: points were rewarded in real time for data input (maximum reward of 21 points per week) as well as to responding to staff follow-up calls (5 points each), attendance of clinic visits (10 points each), and negative anti-HIV test results (50 points each). Points rewarded were part of the intervention and were available to the YFS+APP arm only. Points were exchangeable for cash, with redemption being available at every 100 points if redeemed before the end of the study. Moreover, 100 points were exchangeable for ฿100 (US $3) at month 3 or month 6 clinic visits. A maximum of 719 points could be accumulated when using the app for 6 months. "		
**5-ix) Describe use parameters**		
**5-x) Clarify the level of human involvement**		
**5-xi) Report any prompts/reminders used**		
Users could set prompts to input data to the app if they chose to but this was not mandatory for all users. The details of this were not mentioned in this manuscript as they had previously been detailed in an earlier published manuscript on the same application.		
**5-xii) Describe any co-interventions (incl. training/support)**		
"Participants could be new, current, or former PrEP users. They were randomized (1:1) to receive oral daily tenofovir-disoproxil fumarate/emtricitabine provided by either youth-friendly services (YFS) only or YFS plus the use of the Raincoat app (YFS+APP)."<br><br>"YFS included the following: (1) monthly engagement via either in-person clinic visits (months 1, 3, and 6) or telephone calls (months 2, 4, and 5) and (2) access to counselors and site staff outside scheduled visits through web-based messaging or telephone calls with responses provided within 24 hours. Clinic visits were available during weekdays and Saturday mornings to accommodate adolescent lifestyles. Motivational interviewing focused on HIV risk reduction and empowerment on using available HIV prevention methods was used by counselors during all interactions with clients [35,36]."		
**6a) CONSORT: Completely defined pre-specified primary and secondary outcome measures, including how and when they were assessed**		
"Our aim in this study is to investigate participant engagement and the impact on PrEP adherence of the Project Raincoat mobile phone app, which was based on the IMB skills model developed to support PrEP adherence among YMSM and YTGW at risk of HIV acquisition, in the context of an adolescent-friendly clinic providing bidirectional web-based communications in Thailand."<br><br>"Data on app use were downloaded for analysis after 6 months of follow-up. App paradata collected included first and last log-in dates, number of times logged in, number of LRAs, use of reminder messages, use of the medication alarm function, and total points accumulated.<br>The number of LRAs was used as an independent variable to assess user engagement with the app. TFV-DP DBS concentrations were used to evaluate PrEP adherence, with TFV-DP levels ≥700 fmol/punch considered good adherence (equivalent to ≥4 tablets/week) [23].<br>Continuous variables were presented as means with SDs or medians with IQRs, and categorical variables with absolute numbers and percentages. The chi-square test, Z test, Fisher exact test, odds ratios, and 95% CIs were used for group comparisons and associations as appropriate. Stata/SE (version 13.0; StataCorp) was used for quantitative data analyses."		
**6a-i) Online questionnaires: describe if they were validated for online use and apply CHERRIES items to describe how the questionnaires were designed/deployed**		

**6a-ii) Describe whether and how "use" (including intensity of use/dosage) was defined/measured/monitored**		
**6a-iii) Describe whether, how, and when qualitative feedback from participants was obtained**		
**6b) CONSORT: Any changes to trial outcomes after the trial commenced, with reasons**		
"This study was conducted in two different settings: (1) medical center–based facilities at the Thai Red Cross AIDS Research Center (TRCARC), the largest voluntary HIV testing center of Thailand, and the King Chulalongkorn Memorial Hospital, a major teaching hospital in Bangkok and (2) key population-led community-based drop-in centers operating as satellite sites of TRCARC, namely, the Rainbow Sky Association of Thailand (RSAT) and Service Workers In Group (SWING), both located in Bangkok, Thailand."		
**7a) CONSORT: How sample size was determined**		
**7a-i) Describe whether and how expected attrition was taken into account when calculating the sample size**		
**7b) CONSORT: When applicable, explanation of any interim analyses and stopping guidelines**		
"Our aim in this study is to investigate participant engagement and the impact on PrEP adherence of the Project Raincoat mobile phone app, which was based on the IMB skills model developed to support PrEP adherence among YMSM and YTGW at risk of HIV acquisition, in the context of an adolescent-friendly clinic providing bidirectional web-based communications in Thailand."<br><br>"Data on app use were downloaded for analysis after 6 months of follow-up. App paradata collected included first and last log-in dates, number of times logged in, number of LRAs, use of reminder messages, use of the medication alarm function, and total points accumulated.<br>The number of LRAs was used as an independent variable to assess user engagement with the app. TFV-DP DBS concentrations were used to evaluate PrEP adherence, with TFV-DP levels ≥700 fmol/punch considered good adherence (equivalent to ≥4 tablets/week) [23].<br>Continuous variables were presented as means with SDs or medians with IQRs, and categorical variables with absolute numbers and percentages. The chi-square test, Z test, Fisher exact test, odds ratios, and 95% CIs were used for group comparisons and associations as appropriate. Stata/SE (version 13.0; StataCorp) was used for quantitative data analyses."		
**8a) CONSORT: Method used to generate the random allocation sequence**		
Not applicable to this trial - all participants in this trial were cared for by the same care provider team.		
**8b) CONSORT: Type of randomisation; details of any restriction (such as blocking and block size)**		
"Participants could be new, current, or former PrEP users. They were randomized (1:1) to receive oral daily tenofovir-disoproxil fumarate/emtricitabine provided by either youth-friendly services (YFS) only or YFS plus the use of the Raincoat app (YFS+APP)."		
**9) CONSORT: Mechanism used to implement the random allocation sequence (such as sequentially numbered containers), describing any steps taken to conceal the sequence until interventions were assigned**		
"Participants could be new, current, or former PrEP users. They were randomized (1:1) to receive oral daily tenofovir-disoproxil fumarate/emtricitabine provided by either youth-friendly services (YFS) only or YFS plus the use of the Raincoat app (YFS+APP)."<br><br>Randomisation was via block randomisation (1:1) done by a computer and the sequence of randomisation placed in sealed envelopes opened at point of randomisation.		
**10) CONSORT: Who generated the random allocation sequence, who enrolled participants, and who assigned participants to interventions**		
A computer generated the random allocation sequence<br><br>Research staff enrolled the participants and assigned participants to interventions based on the allocation set by the computer.		
**11a) CONSORT: Blinding - If done, who was blinded after assignment to interventions (for example, participants, care providers, those assessing outcomes) and how**		
**11a-i) Specify who was blinded, and who wasn't**		
Not applicable - it was not possible to blind the intervention used in this study.		

**11a-ii) Discuss e.g., whether participants knew which intervention was the “intervention of interest” and which one was the “comparator”**		
**11b) CONSORT: If relevant, description of the similarity of interventions**		
Not applicable, only one ehealth intervention used in this trial, the comparator group did not have any health interventions		
**12a) CONSORT: Statistical methods used to compare groups for primary and secondary outcomes**		
not applicable - no clustering by care providers in this study		
**12a-i) Imputation techniques to deal with attrition / missing values**		
Those that did not use the app were a specific group used as part of the statistical analysis in this trial.		
**12b) CONSORT: Methods for additional analyses, such as subgroup analyses and adjusted analyses**		
not applicable - no other subgroup analyses performed		
RESULTS		
**13a) CONSORT: For each group, the numbers of participants who were randomly assigned, received intended treatment, and were analysed for the primary outcome**		
"In brief, between March 2018 and June 2019, 200 HIV-negative participants were enrolled, 100 received YFS and 100 YFS+APP. The median age at enrollment was 18 (IQR 17-19) years, with 73.5% (147/200) self-defining as YMSM and 26.5% (53/200) as YTGW. Participants were enrolled and followed-up at medical center–based facilities (135/200, 67.5%) or community-based drop-in centers (65/200, 32.5%). Baseline characteristics between the YFS and YFS+APP arms were similar except for self-reported substance use in the preceding 3 months, which was higher in the YFS+APP arm, 18% (18/100), than in the YFS arm, 8% (8/100; P=.04) [34]. Of the 143 adolescents reporting sexual activity in the past month, 94 (65.7%) reported inconsistent condom use. Of the 200 participants, 187 (93.5%) rated themselves as having a low risk of HIV acquisition. There were no significant differences in PrEP adherence between the YFS and YFS+APP arms. PrEP adherence was 51% (40/79) in the YFS arm and 54% (44/81) in the YFS+APP arm (P=.64) at month 3 and was 44% (30/68) in the YFS arm and 49% (36/73) in the YFS+APP arm (P=.54) at month 6, further details of which have previously been published [34]."<br><br>"Of the 100 participants randomized to receive YFS+APP, 87 (87%) used LRA at least once during the follow-up period. Of these 87 app users, 55 (63%) used their own phones, and the remaining used loaned Android operating system phones. Of the 13 participants who never logged into the app, 10 (77%) used their own phones and 3 (23%) used loaned phones. Among the app users, the median (IQR) duration between the first and last LRA was 3.5 (1.6-5.6) months, with a median LRA frequency of 6 (IQR 2-10). There was no difference in median LRA frequency between participants who used their phones and those who used loaned phones (P=.21). The percentage of participants who used LRA declined over time, that is, 77%, 52%, and 28% at 2, 6, and 12 weeks after enrollment, respectively (Figure 1)."		
**13b) CONSORT: For each group, losses and exclusions after randomisation, together with reasons**		
Not applicable - these details were published previously and not mentioned in this paper. In our previous publication we reported 27% were lost to follow-up overall at 6 months (end) of the trial		
**13b-i) Attrition diagram**		
**14a) CONSORT: Dates defining the periods of recruitment and follow-up**		
"June 2019, 200 HIV-negative participants were enrolled, 100 received YFS and 100 YFS+APP. The median age at enrollment was 18 (IQR 17-19) years, with 73.5% (147/200) self-defining as YMSM and 26.5% (53/200) as YTGW. Participants were enrolled and followed-up at medical center–based facilities (135/200, 67.5%) or community-based drop-in centers (65/200, 32.5%). Baseline characteristics between the YFS and YFS+APP arms were similar except for self-reported substance use in the preceding 3 months, which was higher in the YFS+APP arm, 18% (18/100), than in the YFS arm, 8% (8/100; P=.04) [34]. Of the 143 adolescents reporting sexual activity in the past month, 94 (65.7%) reported inconsistent condom use. Of the 200 participants, 187 (93.5%) rated themselves as having a low risk of HIV acquisition. There were no significant differences in PrEP adherence between the YFS and YFS+APP arms. PrEP adherence was 51% (40/79) in the YFS arm and 54% (44/81) in the YFS+APP arm (P=.64) at month 3 and was 44% (30/68) in the YFS arm and 49% (36/73) in the YFS+APP arm (P=.54) at month 6, further details of which have previously been published [34]."		
**14a-i) Indicate if critical “secular events” fell into the study period**		

**14b) CONSORT: Why the trial ended or was stopped (early)**		
not applicable - trial was not stopped early		
**15) CONSORT: A table showing baseline demographic and clinical characteristics for each group**		
Please see table 1: Baseline characteristics of participants in the YFS+APPa arm who logged in to the mobile phone app and performed risk assessment (LRAb) at least once during the trial follow-up period and associations with LRA.		
**15-i) Report demographics associated with digital divide issues**		
Please see table 1: Baseline characteristics of participants in the YFS+APPa arm who logged in to the mobile phone app and performed risk assessment (LRAb) at least once during the trial follow-up period and associations with LRA.		
**16a) CONSORT: For each group, number of participants (denominator) included in each analysis and whether the analysis was by original assigned groups**		
**16-i) Report multiple "denominators" and provide definitions**		
Please see Table 1, Table 2, Table 3		
**16-ii) Primary analysis should be intent-to-treat**		
**17a) CONSORT: For each primary and secondary outcome, results for each group, and the estimated effect size and its precision (such as 95% confidence interval)**		
"The overall percentage of PrEP adherence was higher in frequent app users than in infrequent app users and the YFS arm, but this did not reach statistical significance. At month 6, the proportion of participants who achieved TFV-DP≥700 fmol/punch was 59% (13/22), 44% (30/68) among frequent app users, 45% (21/47) for infrequent users, and 44% (30/68) in the YFS arm (P=.47)."		
**17a-i) Presentation of process outcomes such as metrics of use and intensity of use**		
**17b) CONSORT: For binary outcomes, presentation of both absolute and relative effect sizes is recommended**		
Please see Table 1, Table 2, Table 3		
**18) CONSORT: Results of any other analyses performed, including subgroup analyses and adjusted analyses, distinguishing pre-specified from exploratory**		
Not applicable - no other subgroup analyses performed		
**18-i) Subgroup analysis of comparing only users**		
**19) CONSORT: All important harms or unintended effects in each group**		
Not applicable - no important harms or unintended effects were seen in this trial.		
**19-i) Include privacy breaches, technical problems**		
**19-ii) Include qualitative feedback from participants or observations from staff/researchers**		
DISCUSSION		
**20) CONSORT: Trial limitations, addressing sources of potential bias, imprecision, multiplicity of analyses**		
**20-i) Typical limitations in ehealth trials**		

"Although the original design of this study was fully powered to compare those who received YFS versus YFS+APP, as a significant proportion of study participants infrequently used the app, it became necessary for the final analysis to stratify outcome groups into frequent users (LRA≥10) and infrequent users (LRA<10). The actual analysis was therefore not fully powered; thus, results from this study should be viewed as preliminary as part of a pilot randomized control trial. Another limitation of this study was that the app used for the intervention in this study was based on just 2 FGDs, which may have provided an inadequate range of views in the design of the app. In addition, the protocol of this study was designed to describe the natural history of app use without any formal encouragement from the study team. App paradata were therefore collated at the end of study for this analysis. However, another possible policy that could have been taken was to continually look at app use and discuss app use obstacles at each contact to rapidly assess and address functionality problems and also to collect design issues real time when these issues are fresh in the minds of the users and more likely to be recalled more clearly. In addition, given that the efficacy of mobile technologies is also linked to literacy and the performance of this study in a large city, findings from it may not apply to a more rural area where access to mobile technologies and literacy may be more challenging and less benefit gained from such a form of service provision enhancement, a limitation of mHealth seen previously [44]. Only a total of 6 interviewees participated in FGDs that informed the design of this app, which may have affected the generalizability of the preference representation drawn from this sample of adolescents. The sampling frame used for exit interviews in this study was also biased toward those who were classified as frequent users of the app, which may have led to findings being more biased toward favoring app functions. Given no differences were seen between arms, interviews with control arm participants could have been done to provide further information on possible reasons for this. Interviews in this study were conducted for quite a long time after study completion in most cases, which may have led to limited data quality owing to implications on participant recall. The small sample size of this study also limits the generalizability of the conclusions drawn from this study, and it is possible that the intervention was not efficacious or the sample size was too small to see any effect present."		
**21) CONSORT: Generalisability (external validity, applicability) of the trial findings**		
**21-i) Generalizability to other populations**		
**21-ii) Discuss if there were elements in the RCT that would be different in a routine application setting**		
**22) CONSORT: Interpretation consistent with results, balancing benefits and harms, and considering other relevant evidence**		
**22-i) Restate study questions and summarize the answers suggested by the data, starting with primary outcomes and process outcomes (use)**		
"This randomized controlled study examined how a smartphone PrEP adherence support app used among adolescents in Thailand affected PrEP adherence and observed higher proportions of PrEP adherence among frequent app users than among infrequent users and the YFS arm at both months 3 and 6, although this did not reach statistical significance. "		
**22-ii) Highlight unanswered new questions, suggest future research**		
Other information		
**23) CONSORT: Registration number and name of trial registry**		
"Trial Registration: ClinicalTrials.gov NCT03778892; https://clinicaltrials.gov/ct2/show/NCT03778892"		
**24) CONSORT: Where the full trial protocol can be accessed, if available**		
Available in the references section of the paper.		
**25) CONSORT: Sources of funding and other support (such as supply of drugs), role of funders**		
"CIPHER (Collaborative Initiative for Pediatric HIV Education and Research; grant 2017/472-SON) provided core funding for this study. The medication and medical care provided through the Princess PrEP Program was supported by the US President's Emergency Plan for AIDS Relief and the US Agency for International Development through the Linkages Across the Continuum of HIV Services for Key Populations Cooperative Agreement (grant AID-OAA-A-14-0045) managed by FHI 360. The Ratchadapisek Sompoch Endowment Fund (2019-2020) under Telehealth Cluster, Chulalongkorn University, provided supplemental mHealth Research Funding Support and also funding for mHealth staff capacity building, The Chulalongkorn University C2F Postdoctoral Fellowship Fund supported SK, and The Chulalongkorn University Ratchadapisek Sompoch Postdoctoral Fellowship Fund (2019-2020) supported WNS."		
**X26-i) Comment on ethics committee approval**		
**x26-ii) Outline informed consent procedures**		

**X26-iii) Safety and security procedures**		
**X27-i) State the relation of the study team towards the system being evaluated**		
